# Large Strangulated Spigelian Hernia: Management of an Uncommon Presentation of Abdominal Hernias in Central Uganda

**DOI:** 10.1155/2019/8474730

**Published:** 2019-10-13

**Authors:** Wasingya Lucien, Franck Katembo Sikakulya, Kisembo Peter, Atwijukire Vincent

**Affiliations:** ^1^Department of Surgery, Kitovu Hospital, Uganda; ^2^Department of Surgery, Kampala International University, Western Campus, Ishaka, Uganda; ^3^Department of Surgery, Université Catholique du Graben, Butembo, Democratic Republic of the Congo

## Abstract

**Background:**

Spigelian hernia is an uncommon presentation of abdominal hernias with 0.1-2%. We report a case of a large strangulated Spigelian hernia, an uncommon presentation of abdominal hernias, and its management in a health facility in Central Uganda.

**Case Presentation:**

A 76-year-old female presented with a 2-day history of colicky abdominal pain, bilious vomiting, and abdominal distension. On abdominal ultrasound scan, an abdominal wall defect measuring 4.45 cm with herniated bowel loops in the left anterior abdominal region with mild fluid collection in the hernia sac was seen. Conservative management for intestinal obstruction which included putting the patient on nil per os, NG tube decompression, and soapy enema was instituted, and surgery was done on the second day of admission. Intraoperatively, using a Rutherford-Morrison incision, we found a large defect at the Spigelian aponeurosis, with an inflamed sac protruding. The Spigelian hernia was repaired with a mesh under layers. The patient recovered uneventfully and was discharged 10 days after surgery.

**Conclusion:**

Clinicians and especially general surgeons might be aware of this rare condition in most of the anterior abdominal swellings. Strangulation is the commonest complication of Spigelian hernia, and surgical management remains the mainstay of its treatment.

## 1. Introduction

**Figure 1:**
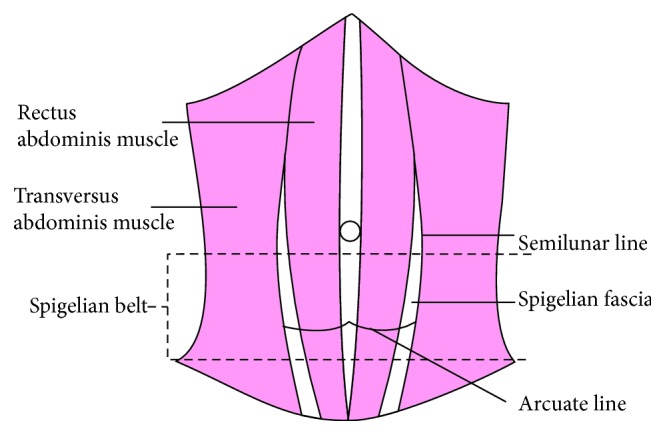
The location of Spigelian fascia and Spigelian belt (the image was drawn by Zhou Ye) [3].

In all abdominal wall hernias, Spigelian hernia (SH) represents a rare surgical condition with 0.1-2%, frequent in patients aged between 40 and 70 years with a predominance in the female gender than male with a sex ratio of 1.18 : 1 [1]. A Spigelian line marks the transition from the muscle to the aponeurosis in the transversus abdominis muscle of the abdomen. It is a lateral convex line between the costal arch and the pubic tubercle [2]. In more than 90% of presentation, Spigelian hernia has been located in the “Spigelian belt,” a transverse 6 cm wide zone in the lower abdominal wall (Figure 1) [3].

The Spigelian hernia is clinically asymptomatic in 90% cases and has nonspecific clinical findings. However, vague abdominal pain can be associated with it. Severe complications marked its evolution such as strangulation in up to 24%, and patients should be offered an immediate surgical management [4, 5].

In this paper, we need to report a case of a large strangulated Spigelian hernia, an uncommon presentation of abdominal hernias, and its management in a health facility in Central Uganda.

## 2. Case Presentation

A 76-year-old female, known to be hypertensive on treatment, presented with a 2-day history of colicky abdominal pain, bilious vomiting, and abdominal distension. On examination, she was sick looking and afebrile.

On physical examination, she had a moderately distended abdomen, with a moderately tender swelling in the left anterior abdominal region which according to the patient had been there for over 20 years. The rest of the abdomen was mildly tender but no rebound tenderness, and bowel sounds were of increased frequency and pitch. A digital rectal exam revealed an empty rectum. Notably from the cardiovascular system exam, she was in hypertensive urgency with a blood pressure of 200/100 mmHg.

**Figure 2:**
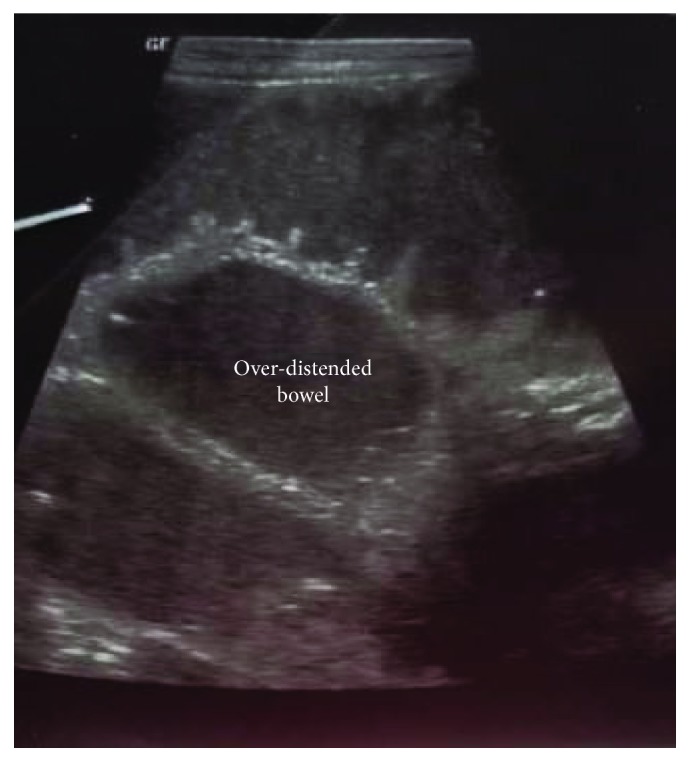
Abdominal ultrasound scan showing the over-distended bowel in the left anterior abdominal wall.

**Figure 3:**
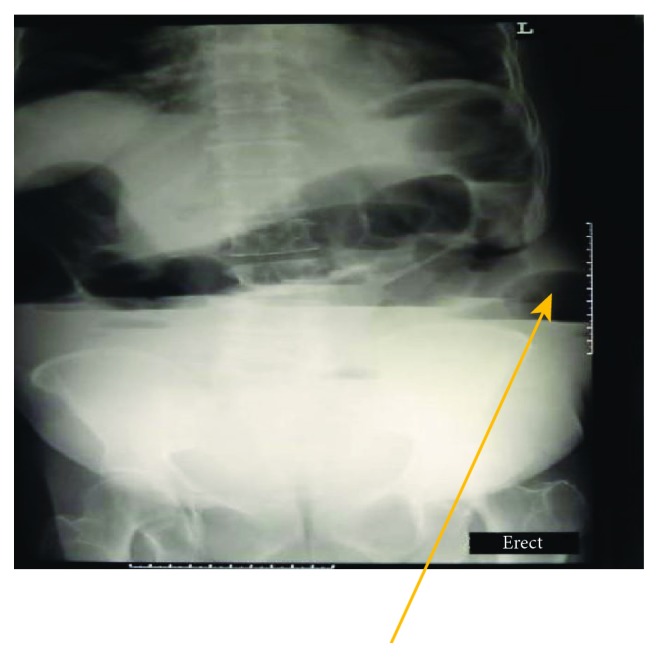
Abdominal X-ray anteroposterior showing air fluid level with a protrusion in the left anterior abdominal wall.

Investigation results show the following data: full blood count: total white cell count 13.9 g/L, hemoglobin 11.9 g/dL, and platelets 563 g/L; serum electrolytes: chloride 99.9 mmol/L, sodium 134.9 mmol/L, and potassium 5.40 mmol/L generally normal; and abdominal ultrasound scan: bowel loops were distended (Figure 2) with contents showing back and forth peristaltic motion. There was also an abdominal wall defect measuring 4.45 cm in diameter with herniated bowel loops in the anterior abdominal region with mild fluid collection in the hernia sac. An erect abdominal X-ray revealed uneven bowel gas distribution mainly in the upper abdomen with dilated bowel loops and significant air fluid levels (Figure 3).

CT scan was requested but not done due to financial constraints. Working impression was intestinal obstruction due to sigmoid volvulus, also querying an obstructed hernia in the left anterior abdominal wall and hypertensive urgency.

Conservative management for intestinal obstruction which included nil per os, nasogastric tube decompression of the stomach, soapy enema, maintenance intravenous fluids, and antibiotic cover was started plus blood pressure management, and 24 hours later, she had improved as the abdominal pain, distension, and vomiting had stopped. She was able to pass stool normally. The blood pressure had also normalized. Decision was made to repair the hernia; and an explanation was given to the patient, and informed consent was obtained.

**Figure 4:**
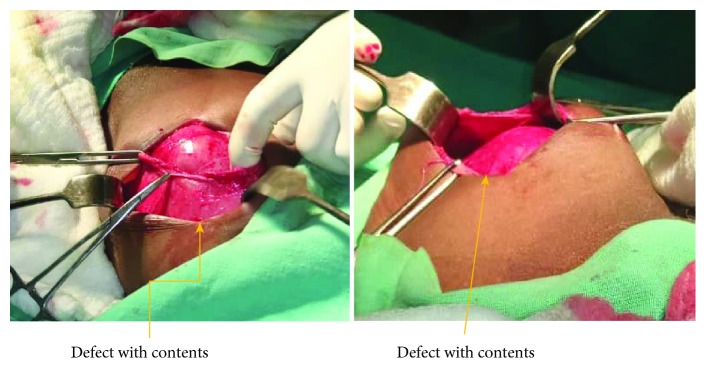
The defect of Spigelian hernia with contents.

**Figure 5:**
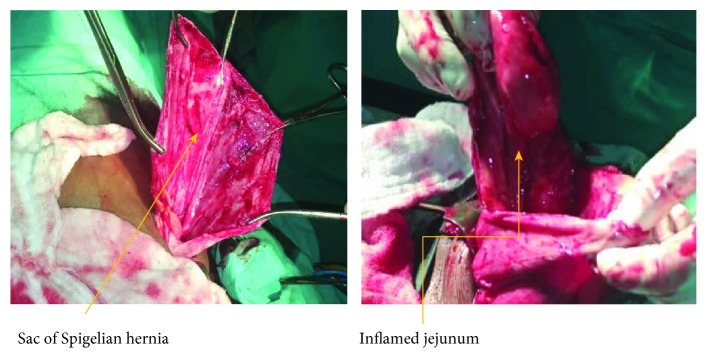
Sac of Spigelian hernia and the inflamed jejunum in a perioperative period.

**Figure 6:**
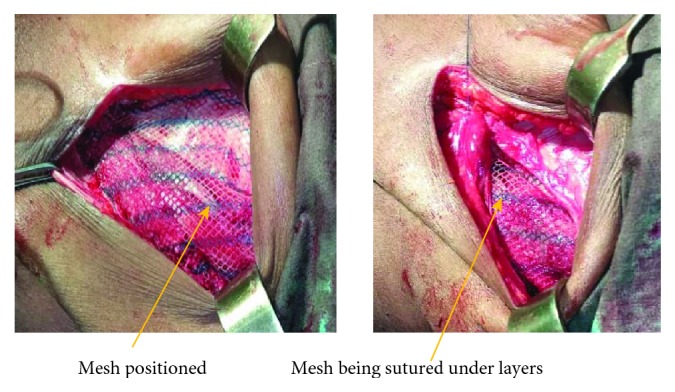
Operating wound showing the mesh being positioned and being sutured under the layers in the left anterior abdominal wall.

**Figure 7:**
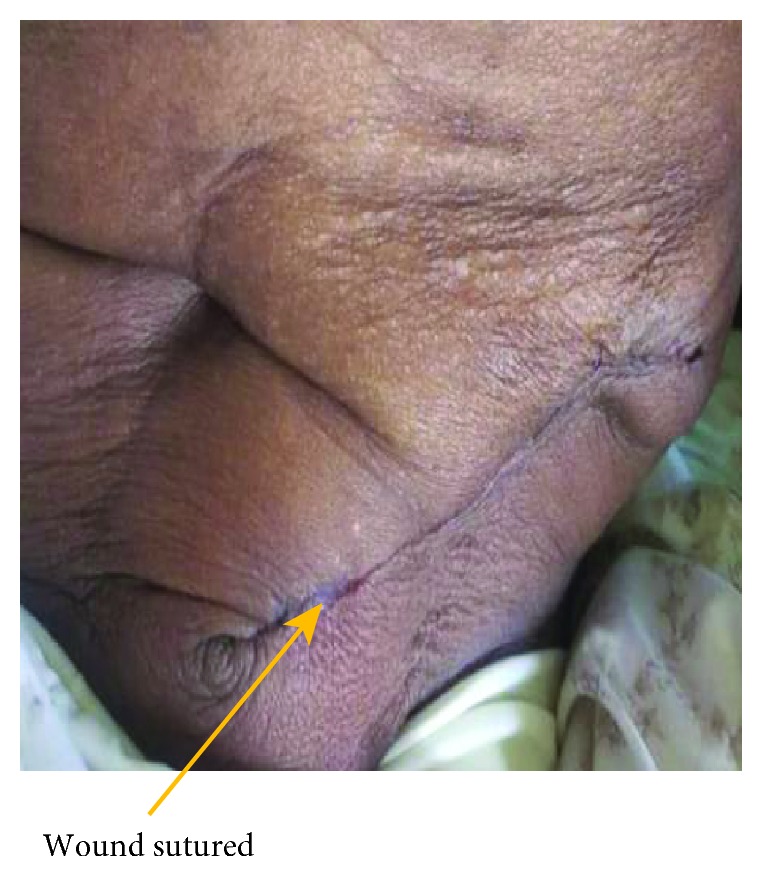
The wound on the anterior abdominal wall after being sutured.

Intraoperatively, using a Rutherford-Morrison incision in the left anterior abdominal region, the abdomen was opened in layers; a large defect was found (6 × 5 cm) at the Spigelian aponeurosis (Figure 4), with a huge, thick, inflamed sac protruding; the sac was opened; and an inflamed jejunum was found, obstructed with adhesions (Figure 5). The Spigelian hernia was repaired with a mesh under layers (of the rectus sheath), fixing it with Nylon 0, from the lower part of the inguinal ligament and up to the sheath of the muscles; the mesh was covered with the sheath of the rectus and transversus muscle (Figure 6). The mesh was subcutaneously apposed with Vicryl 2/0, and the skin was closed with Vicryl 2/0 (Figure 7). The patient recovered uneventfully and was discharged on the tenth postoperative day. The follow-up was done at 2 months and 6 months, and no sign of recurrence was identified.

## 3. Discussion

Spigelian hernia is an uncommon abdominal hernia with 0.1 to 2% of all abdominal defects [1]. It is commonly seen in patients aged between 40 and 70 years as it was in our case report [4]. Even if there is not much substantial difference reported about sex, it has been shown that the incidence of Spigelian hernia is more frequent in females than in males [4]. Abdominal swelling, abdominal pain, and signs of intestinal obstruction are common manifestations of Spigelian hernia [4]. In our case, the 76-year-old female came with the same symptoms mentioned above. The abdominal pain varies from 31% to 86% of cases [6]. The ultrasound scan was our first-line imaging in the investigation of our case as requested in the literature and could be followed by CT scan in challenging cases which was not done due to financial constraints [7].

In 24% of the cases, the strangulation is a major complication of Spigelian hernia as seen in our case report [5]. The contents of the sac are mostly the omentum but can also include the small intestine, colon, stomach, gallbladder, Meckel's diverticulum, appendix, ovaries, and testes [8]. The contents of the sac in our case were inflamed jejunum, obstructed with adhesions. In developed countries and in some well-equipped hospitals in developing countries, laparoscopy is playing a major role in abdominal surgery [9].

In Spigelian hernia, mesh repair associated with laparoscopy correction is the best procedure in developed countries [8], but in our area, we went ahead with open surgery using a Rutherford-Morrison incision for easy access to the defect and good exposure as the hernia was big and strangulated. We corrected the defect using a gold standard technique, mesh repair and fixed the mesh under layers using Nylon 0, from the lower part of the inguinal ligament and up to the sheath of the muscles (Figure 5). In large defects, mesh repair is the best surgical treatment of Spigelian hernia and has good results according to Chaouch et al. [4].

## 4. Conclusion

A Spigelian hernia is really an uncommon presentation of abdominal hernias, but it exists. Its presentation is quite asymptomatic, and its diagnosis is shown by the signs of its complications. Clinicians and especially general surgeons might be aware of this rare condition in most of the anterior abdominal swellings. Findings from supportive investigations play a major role in its diagnosis. Strangulation is the commonest complication of Spigelian hernia, and surgical management remains the mainstay of its treatment.
